# Using Drama Therapy to Enhance Maternal Insightfulness and Reduce Children’s Behavior Problems

**DOI:** 10.3389/fpsyg.2020.586630

**Published:** 2021-01-20

**Authors:** Rinat Feniger-Schaal, Nina Koren-Karie

**Affiliations:** ^1^The Center for the Study of Child Development, University of Haifa, Haifa, Israel; ^2^Graduate School of Creative Arts Therapies, University of Haifa, Haifa, Israel; ^3^School of Social Work, University of Haifa, Haifa, Israel

**Keywords:** drama therapy, parents, intervention, attachment, children at risk

## Abstract

Maternal insightfulness or the capacity to see things from the child’s point of view, is considered to be a crucial construct for therapeutic change. In the present study, we aimed to implement the knowledge gleaned from the studies on attachment theory and maternal insightfulness into clinical practice to create an intervention program for mothers of children-at-risk due to inadequate parental care. We used drama therapy to “practice” maternal insightfulness in more “experiential” ways, because the use of creative expressive means may be accessible and effective for the target population of the study and help improve maternal care. We used a manualized 10-week drama therapy-group intervention, focusing on the core concepts of maternal insightfulness: insightfulness, separateness, complexity, and acceptance. We used various dramatic means to explore and experience these components of maternal insightfulness. Forty mothers of children-at-risk took part in eight groups of parental insightfulness drama therapy (PIDT). To evaluate the efficacy of the intervention, we used the Insightfulness Assessment (IA) interview, which produces 10 scales and a final classification of PI and non-PI. The Child Behavior Check List (CBCL) was used to evaluate a change in children’s behavior problems. The assessment took place at three time points: before the intervention (T1), right after the end of the intervention (T2), and 6 months following the intervention (T3). Results at T2 showed a significant improvement compared to T1 in some of the maternal insightfulness scales, but not in the maternal insightfulness categorical classification. At T3, there was a significant change in the classification of the mothers, from non-insightful to positively insightful. At T3, there was also a significant decline in the children’s externalized and general behavioral problems. The results of this study contribute to an evidence-based practice of using drama therapy in the treatment of mothers and children at risk.

## Introduction

Drama therapy is an active and experimental psychotherapy modality involving the intentional and systematic use of dramatic and theatrical means to achieve psychological growth and change in a psychotherapeutic relationship (Emunah, 2019, http://www.nadta.org). Drama therapy builds on the therapeutic aspects that are present in drama and theater, including creativity, playing, exploration, and role acting (Jennings, 1992; Jones, 2007). At present, drama therapy is in the crucial stage of moving from a clinical report of case studies and vignettes to producing an evidence-based practice using empirical studies (Feniger-Schaal and Orkibi, 2020). The present study joins this important direction by applying the use of drama therapy in the therapeutic work with parents of children-at-risk due to inadequate parental care and systematically evaluating this therapeutic work using reliable and valid assessment tools. We describe the psychological effect of drama therapy intervention, focusing on maternal insightfulness for mothers of children-at-risk.

How can we use drama therapy to help parents who experience difficulties in the relationship with their children? The present paper addresses this question based on data from a research project that involved eight groups that used the parental insightfulness drama therapy (PIDT) model at community welfare parent-child centers (for details of the protocol-based intervention, see masked for review; Feniger-Schaal et al., 2013). The PIDT model makes a connection between drama therapy and attachment theory and between drama therapy practice and developmental psychology. In this model, drama therapy serves as a platform on which to “practice” and experience the parental stance that improves the parent-child relationship. In the presentation of our theoretical background, we describe the core components of attachment theory regarding parental insightfulness and tie it to drama therapy practice.

### Maternal Insightfulness

The parental capacity to see things from the child’s point of view has been the focus of a body of research that involved assessing mothers’ representations of their children and of themselves as caregivers (Fraiberg et al., 1975; Fonagy et al., 1991; Lieberman, 1997). Research on mentalizing has expanded over the past two decades, leading to the development of several similar concepts: parental mind-mindedness (Meins, 1997), parental reflective functioning (Slade, 2005), and parental insightfulness (Oppenheim and Koren-Karie, 2002). All these concepts have been investigated as predictors of sensitive parenting that affect the quality of parent-child relationships (for more details on the various concepts, see Zeegers et al., 2017). One of the main differences between the concpets is the operationalization (the way to assess) of each concept. In the present outcome study, we prioritzed the measurment chosen for assessing the outcomes of the intervention, and we detail below the uniqness of the parental Insightfulness Assessment (IA).

Parental insightfulness^1^ is defined as the capacity of a parent to see things from the child’s point of view and empathically understand the motives underlying the child’s behavior (Oppenheim and Koren-Karie, 2009). It is considered as the internal process that underlies the parent’s capacity to respond sensitively and appropriately to the child’s emotional signals and to enhance secure attachment (Koren-Karie et al., 2002).

Parental insightfulness involves four main features: an insight regarding the motives for the child’s behaviors; an emotionally complex view of the child; separateness from the child; acceptance and openness to new, and at times unexpected or challenging, information regarding the child. Koren-Karie and Oppenheim (2009, 2018) developed the IA interview, which makes possible a deep understanding of parental insightfulness through the assessment of this construct both clinically and in research. In the course of IA, the mother (or father) is shown three video segments taken from interactions with her child and is asked about her perceptions of her child’s thoughts and feelings during each of the segments, as well as about her own thoughts and feelings. This assessment allows observing how parents’ general representations of their children are applied to make sense of a concrete moment in the life of the child (Oppenheim and Koren-Karie, 2009). Interviews are transcribed and coded; the coding process includes rating of 10 scales: Insight, Openness, Complexity, Focus on the child, Richness, Coherence, Acceptance, Anger, Concern, and Separateness. Based on the scores achieved on the 10 scales, respondents are classified into four groups, one positive insightfulness (PI) category and three non-positively insightfulness (non-PI) categories: one-sided, disengaged, and mixed.

Koren-Karie and colleagues showed that maternal insightfulness is associated with sensitive maternal behavior and secure attachment of the child (see also Koren-Karie et al., 2002; Oppenheim et al., 2009; Oppenheim and Koren-Karie, 2009; Yuval-Adler, 2010; Koren-Karie and Oppenheim, 2018; Feniger-Schaal et al., 2019). Similar results were found in a study that involves fathers (Marcu et al., 2016). The explanation for these associations is thought to be that parental insightfulness promotes relationships that engender the feeling in children that their inner world is meaningful and that their thoughts and feelings are appreciated, understood, and accepted.

Bowlby (1988) claimed that the emotional and behavior problems of young children are rooted in a parenting that lacks empathy toward the child’s basic need for protection and lacks insightfulness into the motives underlying the child’s behavior. Hence, parental insightfulness, sensitive parenting, secure attachment, and child’s behavior problems are closely intertwined.

The idea that a parent’s lack of insightfulness or failure to empathically understand the motives and emotional needs underlying the child’s behavior may be at the root of children’s behavior problem is not only based on Bowlby’s (1988) writings but has received empirical support as well. Oppenheim et al. (2004) examined the connection between the lack of maternal insightfulness and children’s behavior problems in a sample of 32 clinically referred preschoolers. Mothers and children had been involved (separately) in a 7-month treatment program. The mothers’ treatment consisted of 7 months of one-on-one therapy focusing on the parent-child relationship. Results showed that the behavior problems of children of mothers who shifted from non-PI before treatment to PI after treatment decreased; by contrast, the behavior problems of children of mothers who did not make such gains increased (Oppenheim et al., 2004).

Other studies have used the insightfulness interview to assess and capture the change followed by intervention programs for parents of children with various problems. For example, Siller et al. (2012, 2018) found maternal insightfulness to be a moderator that predicts changes in parental responsive behavior following the treatment for parents of children with ASD. Another study that examined the clinical significance of maternal insightfulness found that children who were exposed to violence exhibited elevated levels of behavior problems only when their mothers were non-PI, emphasizing the connection between children’s behavior problems and maternal insightfulness (Gray et al., 2015). Enhancement of parental insightfulness can therefore be a significant step toward therapeutic change for parents and their children. In the present study, we used a model specifically designed to promote maternal insightfulness using drama therapy, aiming for mothers who experienced difficulties in their relationship with their children.

### Connecting Drama Therapy and Maternal Insightfulness

A central aspect in all art therapies is the possibility of using art to represent externally the inner reality. Thoughts, feelings, emotions, and ideas can be represented by objects, pictures, stories, roles, scenes, etc. In drama therapy, **dramatic projection** (Jones, 2007) is a fundamental process by which participants project an aspect of themselves or their experience onto theatrical materials, thereby externalizing their inner reality into a dramatic reality (Pendzik, 2006). The projection enables a dialogue to take place between the participants’ internally held situation or material and the external expression of that situation or material (see, for example, Feniger-Schaal, 2016). In this way, participants can develop the “audience” aspect of themselves toward their experience, enhancing their ability to engage differently with their materials (Jones, 2007, 2008). Parental insightfulness emphasizes reflective functioning, hence thinking about the mental world of oneself and others (Bateman and Fonagy, 2004). Externalizing the inner emotional states using dramatic means, such as dramatic projection, may foster an expansion of these parts by giving a “stage” that emphasizes it. For example, when mothers in the PIDT program are asked to create a body sculpture to represent their feelings regarding their son’s behavior, they must first think: What are my feelings toward my son’s behavior? Next, they search for an image or metaphor to describe these feelings, which requires another level of “inner search” or “mentalization” (Bateman and Fonagy, 2004). In this way, the dramatic representation itself enhances the need to reflect and to “mind the other’s mind.”

The use of **role-playing** and role taking is another core component of drama therapy (Jones, 2007; Landy, 2009), which seems to be particularly linked to attachment theory, in general (Haen and Lee, 2017), and to parental insightfulness more specifically. When acting as a different character, one must first think about that character as a separate person from one’s own personality, emotions, and desires and then use this understanding of what motivates the character (Noice and Noice, 2006). Goldstein and Winner (2012) described the practice of role-play and pretense in drama classes (by comparison to art classes) as enhancing empathy and the theory of mind and promoting the ability to think about mental states of others and of oneself. The core idea of parental insightfulness is the ability to see things from the child’s point of view. Enacting the role of one’s child demands stepping into his/her shoes and trying to think what he/she feel, what motivates him/her, etc. This process of dramatic action can serve as a powerful tool for enhancing maternal insightfulness.

**Play and playfulness** are other central components of drama therapy (Jones, 2007). Play is a broader term that includes joy, spontaneity, negotiation of needs, allowing a space for “as if” reality, and becoming connected to the inner child (Barnett, 1991; Youell, 2008). These components of play seem strongly related to positive parenting (Menashe-Grinberg and Atzaba-Poria, 2017); therefore, we assume that drama therapy can be an effective vehicle for working with parents who experience difficulties in the relationship with their children. The experience of enjoyment and fun when practicing playfulness in a drama therapy session may be valuable in itself and support the parents’ ability to connect to their children.

In the model used in this study, we used drama therapy to “translate” the ideas of maternal insightfulness into dramatic actions. The idea in using the model is that mothers can experience maternal insightfulness by dramatic means, rather than just talking about it, thus to benefit from the unique qualities of drama therapy mentioned above, that may make the notion of maternal insightfulness more approachable and lively for them. The study has the potential to contribute to the knowledge on the use of drama in therapy by using a clear protocol for intervention, followed by a systematic assessment of the psychological effect of PIDT intervention on mothers and children at risk. We hypothesized that mothers will show improvement in their maternal insightfulness following the PIDT intervention and that the behavior problems of children of mothers who show gains in insightfulness will decrease, whereas the behavior problems of children of mothers who do not show gains will show no change.

## Materials and Methods

We conducted the PIDT program in eight groups in community welfare centers for children and their families, with four to five participants in each intervention group. These centers target children with behavior and emotional problems due to inadequate parental care. Mothers who participated in the PIDT groups were referred to the child-parent centers by social services in the community and were identified as having children with emotional and behavior problems, which was assumed to be related to difficulties in the mother-child relationship. Data were collected at three time points: T1 (before the intervention began), T2 (right after the end of the 10-week intervention), and T3 (6 months after the end of the intervention).

### Participants

The inclusion criteria for participation in the study were referral by social services to the parent-child center; fluency in Hebrew; no major psychiatric diagnosis; and no abuse of drugs or alcohol; no major cognitive impairment. Inclusion criteria for the children were typically developing children aged 5–12 (age of children in the parent-children welfare centers). Only mothers took part in the groups because, in most of the families, the fathers were not involved in therapy, for various reasons. Participants were all from Jewish origin, coming from different geographical areas in Israel.

Sixty mothers were referred to take part in the groups. The final sample at T1 and T2 included 40 mothers who attended the full intervention. Dropouts were mostly before the intervention began (not willing to participate in the group) and at the early stages (meetings 1 or 2). Reasons for dropout were personal difficulties committing to the group, which was defined as a “close group” that demanded the full commitment for the entire 10 sessions. At T3, we were able to contact 32 mothers. The reason for dropout in the final stage had to do with the mothers’ personal difficulties to come to the additional follow-up assessment meeting (although we offered a financial reward for participating in the final assessment).

Participants’ mean age was 37 years (SD = 5.90, range 24–50), and the mean number of years of education was 12.4 years (SD = 1.93, range 7–20). The mothers had on average 2.12 children (SD = 1.22, range 1–6); 50% had a boy and 50% a girl treated at the center; 35% of mothers were married, 42.5% were divorced; and 18% were single parents. The mean age of the children was 8.5 years (SD = 1.95).

### The Intervention

Parental insightfulness drama therapy (masked for review; Feniger-Schaal et al., 2013). The model is a 10-week group intervention for parents of children-at-risk. The aim of the group was to let mothers experience and explore creatively and in a playful way the various components of maternal insightfulness so that they become involved with the insightfulness stance. The intervention focused on parents because of the conviction that the parent has more ability to change the relationship than the child does (van IJzendoorn et al., 1992; Powel et al., 2007). Furthermore, we sought to provide a context for participants to enhance their maternal insightfulness when not under the pressure of reacting to the child; therefore, the groups were for mothers without their children. At the same time, we used dramatic means to make the children present even in their absence, with the mothers role-playing their children.

Group sessions lasted 90 min and were facilitated by two therapists, making it possible to demonstrate “insightful” relationships between the facilitators. Additionally, it was the function of the facilitators to hold and maintain the dramatic space and, thereby, to model and foster the ability to play and be in the dramatic reality.

In the PIDT model, the first four meetings focus on the core elements of maternal insightfulness: insightfulness, separateness, complexity, and acceptance – using various drama therapy techniques to practice and experience each concept. During the six subsequent meetings, the group continued to practice the building blocks of maternal insightfulness to deepen the understanding of the relevance of these concepts to the mother-child relationship. All meetings followed a sequence consisting of a warm-up, the main activity, and closure (a detailed description of the model can be found in masked for the review; Feniger-Schaal et al., 2013).

#### Therapists

All groups were facilitated by two therapists: a trained drama therapist and a “local” therapist who was part of the team of the welfare center. All therapists underwent a 3-day training on the PIDT model. For purposes of fidelity, all therapists wrote a weekly report, and all sessions were video-recorded and observed by the researchers. All therapists received supervision from the first author, who is a trained drama therapist and a registered supervisor, once every 2 weeks.

### Procedure

The study received ethical approval from the Israeli Ministry of Welfare and the IRB committee of the University of Haifa (no. 062/11).

Social workers randomly referred the mothers to the PIDT group and to other groups in the center. The referred mothers were approached by the clinical team at the center for an initial intake. Next, mothers were provided with the necessary information about the intervention and the study. After the participants signed a consent form, their details were given to the study team to schedule the first meeting. Before the intervention (T1), all mothers completed a demographic questionnaire and the CBCL. All the mothers participated in the IA interview, which was recorded and later transcribed. The intake meeting took place mostly at the home of the participant, except for rare cases in which the participant preferred meeting at the welfare center. At the end of the intervention (T2), all mothers participated in the IA interview again. Six months after the intervention, we contacted all the participants and met with them to administer final IA and CBCL. During the 6 months following the intervention, the mothers and their children received no additional psychotherapeutic intervention from the welfare services and reported having had no additional treatment during that time.

### Measures

#### Insightfulness Assessment

The IA is a semi-structured interview that produces continuous scores of 10 scales and a categorical score of four classifications (Oppenheim and Koren-Karie, 2002). The interview consisted of two steps. First, mothers are videotaped interacting with their children in three play episodes (in the present study, the episodes included a competitive game named Jenga, playing with play dough and completing a joint story telling task). Second, mothers are shown the first 2-min segments of each of these episodes. Mothers are then interviewed with regard to each of the video segments and asked what they thought “went through their child’s head,” whether the behaviors observed were typical; and how they felt when they were watching the segment; and whether their child’s behaviors surprised them, concerned them, or made them happy. At the end of the interview, mothers are asked two general questions about their children’s main characteristics and about what struck them most about their children. Throughout the interview, the mothers are asked to illustrate their statements with examples from the observation and from everyday life. Interviews lasted about 45–60 min.

#### Coding Maternal Insightfulness

The interviews were transcribed verbatim, and all identifying information was removed from the transcripts (including the names of interviewees and the time of the interview) (Koren-Karie et al., 2002). The IA produces both dimensional scales and categorical classifications. The 10 scales range from 1 to 9. High scores indicate high levels of the behavior tapped by the scale. In all scales, except for the hostility, concern, and separateness scales, high scores reflect positive behaviors. Rating is done by marking indicators of the various scales as they appear throughout the transcript and then assigning a score on each of the scales based on both the frequency and the strength of the indicators. The rating scales serve as a basis for the classification of the transcripts into one PI and three non-PI categories. Categories reflect more than a simple summation of scale scores. Rather, the coding manual provides guidelines regarding various constellations and combinations of scale scores that lead to each of the specific categories.

The four categories are described below:

1. **Positive insightfulness (PI):** Mothers are characterized by their ability to see various experiences through their child’s eyes and to try to understand the motives underlying their child’s behavior. They are open to the observations of the child on the video segments and may gain new insights as they talk. PI mothers convey an accepting and multi-dimensional picture of their child, and their speech is coherent.
2. **One-sided (OS):** Mothers are characterized by a unidimensional view of their child. They seem to have a preset conception of the child that they impose on the videotaped segments, and this conception does not appear open to change. One-sided mothers tend to switch the focus of discussion from their child to their own feelings or to other irrelevant issues.
3. **Disengaged (DE):** Mothers are characterized by a lack of emotional involvement during the interview. Their answers are short and limited, and they do not use the observation as an opportunity to reflect on their child’s and their own behavior.
4. **Mixed (MX):** This category involves mothers who do not show one type of speech, as defined in the above categories. For example, a mother may sound overwhelmed or hostile in her response to one video segment and uninvolved and uninterested in her response to the other segments.

Insightfulness Assessment validity was shown in numerous studies (Zeegers et al., 2017; Koren-Karie and Oppenheim, 2018). Several studies have used the IA to show the link between mothers’ insightfulness and children’s attachment to their mothers in both normative (Koren-Karie et al., 2002) and high-risk populations (Oppenheim et al., 2009; Feniger-Schaal et al., 2019); some other examples include studies that have used the IA to show the associations between maternal stress (Martinez et al., 2018), maternal childhood trauma, and maternal insightfulness (Koren-Karie and Oppenheim, 2018; Ziv et al., 2018). Another example was shown in a study about mothers’ synchrony behaviors with their children with ASD pre- and post-intervention that was found to be related to the mothers’ insightfulness assessed using the IA. All the studies that used the IA showed good interrater reliability ranging from 0.71 to 0.81 with *p* ≤ 0.01 (for example in Koren-Karie et al., 2002, interrater reliability for the four-way IA classification system was assessed with kappa and was 0.71; *p* ≤ 0.01), and 0.64–0.93 for the ICC calculation of the scales reliability.

The IA coding in the present study was conducted by two independent, experienced, certified coders (the second author and a graduate student), who were blind to any other information about the child, the mother, or the time of evaluation.

Interviews were first coded on ten 9-point scales and yielded the following inter-rater reliabilities, calculated using ICC^2^ (presented in parentheses): Insight into the child’s motives (0.62); Openness/flexibility of thought (0.87); Complexity in description of child (0.85); Maintenance of focus on child (0.77); Richness of description of child (0.73); Acceptance of the child (0.70); Anger (0.88); Concern (0.78); Separateness from child (0.67); and Coherence of thought (0.71). Based on her discourse during the IA, each mother was assigned to one of four insightfulness categories, one category showing PI and three showing non-PI. Kappa^3^ for inter-rater reliability of the IA classifications was 0.73 (*p* < 0.001). Disagreements were resolved by discussion until the consensus was reached.

#### Child Behavior Checklist

The Child Behavior Checklist (CBCL) is a widely used standardized measure of children’s behavior problems (Achenbach, 1991). We used it to obtain the mothers’ reports about the children’s behavior problems. The CBCL contains 112 questions that represent a wide range of behavior problems (e.g., “temper tantrums or hot temper”). For each item, the respondent is asked to indicate the extent to which the item characterizes the child on a 3-point scale, ranging from 0 (not at all) to 2 (very true or often true). The questions in the CBCL are divided into eight scales, which are grouped into three indices: (a) internalizing behavior problems, which include the scales for anxiety/depression, withdrawal/depression, and somatic complaints; (b) externalizing behavior problems, which include the scales for rule breaking behavior and aggressive behavior; and (c) general problems, which include all the previously mentioned scales in addition to social problems, thought problems, and attention problems. The scores were computed using a designated software following the guidelines provided by Achenbach, (1991). A detailed description of its psychometric properties can be found in Nakamura et al. (2009). The CBCL was used in assessments at T1 and T3 (not T2) based on the assumption that because the intervention targeted mothers directly, without their children, the effect on the children’s behavior may reveal itself only at a later stage.

## Results

### Preliminary Analysis

#### Mothers’ Background Information

No significant differences were found between IA classification and mothers’ background information, including the numbers of years of education, age, the number of children (based on Pearson correlation test), and marital status (based on Chi-square test). No associations were found with mothers’ background information and CBCL scores (based on Pearson correlation test and Chi-square test).

#### Children’s Background Information

No significant difference or correlations were found between mothers’ IA and the children’s background information, including the child’s age and gender (using Chi-square test). No associations were found between the children’s CBCL scores and other background information about the child, including gender and age (based on Pearson correlation test and Chi-square test).

#### Distribution of IA Classifications at T1, T2, and T3

Table 1 presents the distribution of maternal insightfulness classification at baseline (T1), post-intervention (T2), and 6 months after the intervention (T3).

**Table 1:** Maternal insightfulness classification.

Maternal insightfulness classification	T1	T2 Frequency (*n*) Percent	T3
Positive insightfulness (PI)	12	19	21
	30%	47.5%	65.6%
One-sided (OS)	26	18	8
	65%	45%	25%
Disengaged (DE)	2	2	2
	5%	5%	6.25%
Mixed (MX)	0	1	1
		2.5%	3.12%
Total (N)	40	40	32

#### Differences in Maternal Insightfulness Between T1 and T2

To test the change in maternal insightfulness, we grouped the maternal insightfulness classification into insightful and non-insightful due to the small number in each of the non-PI categories. The McNemar nonparametric test, which indicates the significance of change in a “before and after” design (Siegel, 1956), showed that an improvement in maternal insightfulness between T1 and T2 was non-significant (*p* = 0.11).

Therefore, we examined the differences in the scales of maternal insightfulness to search for directions of change. Table 2 presents the result of a paired t-test of maternal insightfulness scales to show the difference between T1 and T2. Because of the direction of the change for which we were searching, we used a one-tailed calculation.

**Table 2:** Maternal insightfulness scales at T1 and T2.

Maternal insightfulness scales	T1	T2	*t*	*p*
	M, SD	M, SD		
Focus	4.88, 1.89	5.65, 2.10	−2.83	**0.00**
Insight	4.20, 1.41	4.48, 1.57	−0.91	0.18
Acceptance	4.48, 1.86	5.06,1.76	−1.63	**0.05**
Hostility	2.52, 1.90	2.15, 1.54	1.25	0.11
Concern	5.15, 2.20	3.98, 2.08	3.39	**0.00**
Separateness	6.51, 1.73	6.85, 1.64	−1.12	0.13
Openness	5.02, 1.73	5.30, 1.78	−0.86	0.20
Richness	4.38, 1.44	4.85, 1.54	−1.67	**0.05**
Coherence	3.88, 1.38	4.40, 1.41	−1.93	**0.03**
Complexity	4.73, 1.73	5.12, 1.55	−1.25	0.11

The results show that all the maternal insightfulness scales improved from T1 to T2, half of them showing a statistically significant change.

#### Differences in Maternal Insightfulness Between T2 and the Follow-Up Assessment at T3

To test the difference between T1and T3 in maternal insightfulness classification, based on all 10 scales, we performed a McNemar nonparametric test, which showed that the improvement in maternal insightfulness classification between T1 and T3 was significant (*p* = 0.003). Further analysis, using a paired *t*-test showed a significant change in all the maternal insightfulness scales from T1 to T3 (see Supplementary Material for the scores of the scales and the difference between T1 and T3).

#### Difference in CBCL Between T1 and T3

Table 3 shows the difference in CBCL scores between T1 and T3, as revealed by a paired *t*-test.

**Table 3:** Children’s CBCL scores.

	T1		T3			
	M	SD	M	SD	*t*	*p*
Internalized problems	59.41	10.53	57.21	10.79	2.05	0.281
Externalized problems	60	9.18	56.55	8.70	2.06	**0.048**
General problems	60.24	9.66	56.69	9.30	1.09	**0.049**

*N* = 28, bold represents *p*≤0.05.

The results show a change in children’s behavior problems from T1 to T3. A significant decrease occurred in children’s externalized and general problems but not in children’s internalized problems.

#### Do Changes in Children Parallel Changes in Mothers?

**Figure 1:**
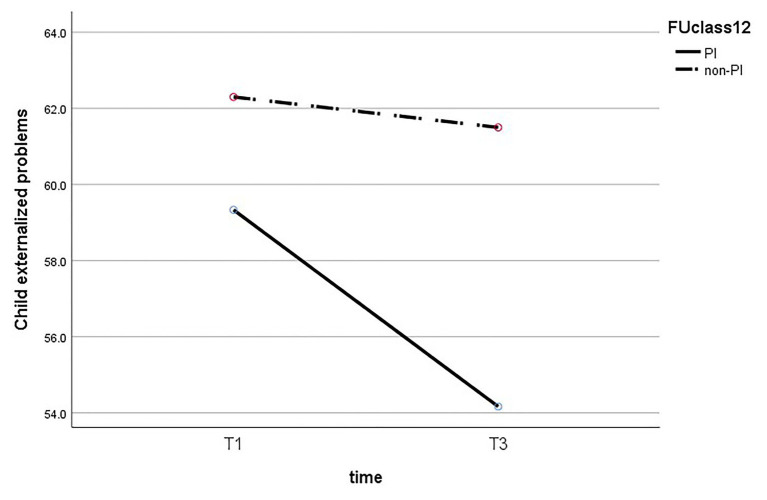
Change in child’s the externalized problems, comparing PI to non-PI mothers.

**Figure 2:**
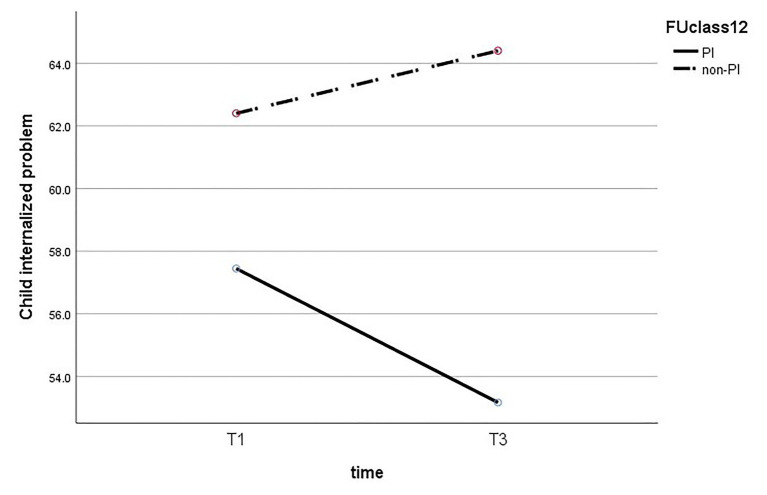
Change in children’s internalized problems, comparing PI to non-PI mothers.

**Figure 3:**
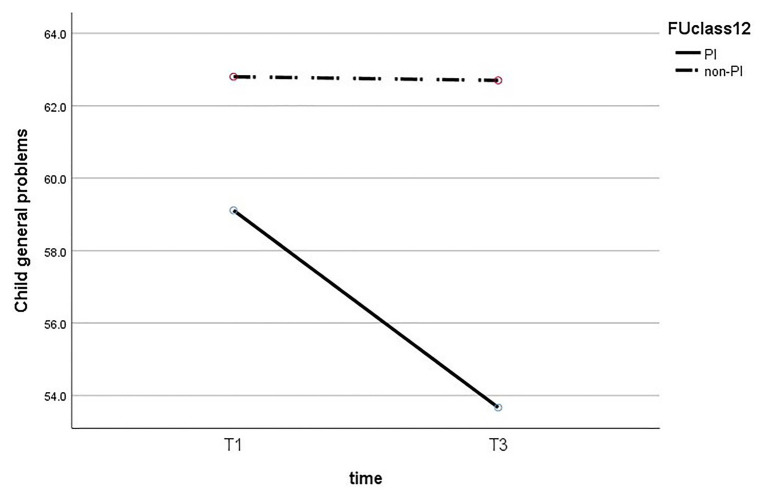
Change in the child’s general problems comparing PI to non-PI mothers.

Next, we performed a mixed ANOVA to test the effect of time and maternal insightfulness on children’s behavior problems. The results reveal that there is no statistically significant interaction between maternal insightfulness and time for internalized problems (*F*_(1,26)_ = 2.195, *p* = 0.151), externalized problems (*F*_(1,26)_ = 1.42, *p* = 0.244), and general problems (*F*_(1,26)_ = 2.153, *p* = 0.154). The results of the ANOVA are presented in Figures 1–3.

In addition, we performed a *t*-test to examine the difference between children’s behavior problems of PI mothers and of non-PI mothers at T3, and found it to be significant for all three behavior problems. The results show a significant difference in children’s internalized problems (*t*_(26)_ = −2.93, *p* = 0.007), so that the scores of internalized problems of children of PI mothers (M = 53.15, SD = 0.04) were lower than those of the children of non-PI mothers (M = 64.4, SD = 10.87). A similar pattern was found for children’s externalized problems (*t*_(26)_ = −2.23, *p* = 0.031), so the scores of children of PI mothers (M = 54.16, SD = 6.09) were lower than those of children of non-PI mothers (M = 61.5, SD = 11.06). Finally, a significant difference was also found in children’s general behavior problems (*t*_(26)_ = −2.38, *p* = 0.033), so that the scores of children of PI mothers (M = 52.66, SD = 6.91) were lower than those of children of non-PI mothers (M = 62.7, SD = 10.81).

## Discussion

In this study, we used a manualized drama therapy-group intervention for mothers of children-at-risk and examined its effect in a systematic way. We hypothesized that an intervention aiming to enhance maternal insightfulness produces benefits for both the mother and the child. As expected, the results show that a 10-week focused drama therapy group resulted in a significant change in both maternal insightfulness and children’s behavior problems. This change, however, unfolded over time. The assessment at the end of the intervention showed no significant change in maternal insightfulness classification. In a follow-up assessment, 6 months after the intervention ended, there were significant changes in both maternal insightfulness and children externalized and general behavior problems.

Mothers’ classification did not change significantly at the end of the intervention, but when examining the maternal insightfulness scales, mothers’ representations were beginning to change in the desired direction, with some of the scales showing a statistically significant change. Focusing on the scales reveals detailed information that may have clinical implications for planning and evaluating therapeutic change. For example, in a previous study, it was found that the mothers’ ability to produce a coherent narrative, measured by the coherence scale, was one of the predicting factors for positive therapeutic gains (Oppenheim et al., 2004). By contrast, the lack of therapeutic change in one of the scales, as measured in our study on the complexity scales, calls attention to how to promote the mothers’ ability to produce a complex and balanced narrative about their child. The lack of change in the hostility scale probably reflects the fact that the score for this scale was not high in T1; therefore, there was not much place to demonstrate a significant change. In general, the lack of change in all the scales right at the end of the intervention represent the lack of overall change in maternal insightfulness, that may have needed a longer time to take place. Maternal insightfulness concerns with the relationship with the child; therefore, the change may have needed time to practice this “mutual dance.” Mothers needed time to practice their new perceptions and behavior with the child and to get a positive response that will reinforce the different way of relating to the child.

Thus, more significant changes were found in the follow-up assessment, 6 months after the intervention ended, when the mothers’ classification on maternal insightfulness (based on all 10 scales) changed significantly, so that nine mothers changed from being non-PI before the intervention to being PI. These results indicate that at this time point more mothers were able to talk about their child without being overwhelmed by worries, negative attributions, and other irrelevant issues. They were able to focus on their child as the center of discussion, expressing sensitivity to the child’s thoughts and feelings. At the same time, they were better able to adopt a complex view of their children and describe them in a balanced way that takes into consideration the children’s strengths and weaknesses. Mothers were also able to see things from their child’s perspective even when their child’s behavior was difficult or contradicted their expectations.

This change can be illustrated by an example from a pre- and post-interview with one of the mothers. In the pre-intervention assessment, the mother watched a 2-min segment of the play episode and was asked about her daughter’s thoughts and feelings during that segment. This is what she said:

“Yes… she was happy to play but at the same time she was impatient. It can also happen in a middle of a conversation; she can suddenly walk away. For example, I spend the whole day with her and she can come up with sentences like “where’s daddy? I want daddy” and like… and then, when he comes home, it’s difficult for her to reunite with him. Really. It takes her a lot of time and sometimes she does not even relate to him the whole evening. For sure, he tries and tries to play with her, but we know that she has this attitude, when she does not want something, kind of snobbish, but it really just looks snobbish, I think it may reflect her insecurity, she does not feel at ease… It’s monotonous for her because I’m always there with her, and this is great because now he’s the one that gets up with her in the morning and makes sandwiches for her for school, so she knows that I’m there and if she will need something she will call me, but now she does not call me. She calls him. She likes that routine, but sometimes she does not want him and she comes to me with a sad face…”

In this pre-intervention assessment, the mother was classified as a one-sided non-PI. As the excerpt illustrates, her response to the interview question was incoherent, full of irrelevant stories, with shifts of focus from the topic at hand to her own thoughts and feelings. It was difficult to obtain a picture of who the child was and why she behaved the way she did. This emotional state pervaded the interview and left no room to consider the child’s thoughts, feelings, and the motives underlying her behaviors.

During the post-treatment assessment, the mother’s responses to the IA questions were markedly different. At this time, her transcript was classified (by a coder who was blind to her first classification) as showing insightfulness. When asked about her child’s thoughts and feelings during the Jenga play, she said:

She was very independent. That was great! She decided what to do, she was looking at what I was doing, but she did not need any help. She felt confident, showed initiative and independence. There is also some kind of maturity here. She’s focused on what she’s doing. I think she wanted us to be able to complete several rounds of this play. She likes to do things that she likes several times in a row.

In the above excerpt, the mother was able to maintain the focus on her child’s thoughts and feelings, her speech was coherent, accepting, and focused.

This change in the mothers’ insightfulness had to do with the change in the children’s behavior problems. Six months after the intervention, the behavior problems of children whose mothers were classified as PI were significantly lower than those of children whose mothers were classified as non-PI. This finding resembles the results of a previous study that found a decrease in the behavior problems of children whose mothers gained PI and an increase in the behavior problems of children whose mothers showed no maternal insightfulness at the end of a therapeutic intervention (Oppenheim et al., 2004). Our results reinforce the notion of reciprocal relations between maternal insightfulness and children’s socioemotional problems. Mother’s ability to see her child’s inner world and keep it in mind made a change in their relationship with the child, and as a result, reducing the children’s behavior problems.

Using drama therapy may have helped mothers change their insightfulness and enhances their ability to see things from the child’s point of view and talk about it in an open and complex way. Using drama to invite the mothers to get into the child’s role and to physically and mentally “sit” in the child’s place may enable the mothers to think about their children as separate persons, who have their own needs and emotional world. Using different projective objects to represent “externally” the child and the relationship “forced” the mothers to broaden their perception of their children and think about them in a multidimensional and complex way. Experiencing empathic acceptance, possibly for the first time in their life, may be the crucial point from which the mothers can develop an accepting and containing relationship with their children and serve as a “secure base” for them.

The changes in children’s behavior problems suggest that the mothers not only changed their inner narrative but were also able to expand and alter their behavior repertoire with their children, which in turn affected the children’s socioemotional state. This change did not appear immediately when the intervention ended but rather had a “sleeper effect”; hence, it took some time to manifest (Bakermans-Kranenburg et al., 2003). As the intervention focused on the representation level of the mother, the change in children’s behavior may not happen at the same time. Changes in the mother’s representations and her behavior must be “accepted” by the child. This involves two people; therefore, it may take time for the change in the mother to obtain a response from the child and for the change to consolidate and affect the mother, the child, and their relationship. This type of belated effect may characterize a therapeutic change in interventions that focus on deeply rooted relational or representational characteristics (Bakermans-Kranenburg et al., 2003) and justify the use of a follow-up assessment to capture the associated changes (Bernard et al., 2017).

The results of the study point to the possibility of using drama therapy in a structured short-term intervention, based on a protocol focused on a clear conceptualization. Most importantly, it stresses the possibility for the use of a systematic and valid assessment to evaluate the contribution of drama-based intervention for psychological change. The present work adds to the scarce literature, demonstrating the empirical contribution of drama therapy to psychological change (Feniger-Schaal and Orkibi, 2020). As such, the study supports the possibility of developing an evidence-based drama therapy practice.

The building blocks of drama therapy are the invitation to play, to be creative, to explore different roles, to pretend, to imagine, and to use metaphors and symbols (Emunah, 2019; Jones, 2007). In the PIDT model (Feniger-Schaal et al., 2013), we sought to use the core elements of drama as a vehicle to enhance maternal insightfulness. The possibility of exploring parenting and the relationship with the child from a playful mindset, which involves not only the mind but also the body, voice, and imagination, enabled the mothers in the group to have a significant and rich experience. This experience embraced the objective complexity of what the mothers had to deal with and, at the same time, invited them to experience parenting as a creative, vivid act.

One of the main characteristics of dramatic reality (Pendzik, 2006) is its ability to hold on to reality and imagination, to the virtual and the concrete, at the same time. In the PIDT model, within the dramatic reality, it was possible to represent the children and the relationship with them dramatically and, in this manner make their presence felt in the group in a way that allowed it to be played with and explored.

Applying drama therapy as an “action method” involves a unique engagement with personal materials, which may lead to therapeutic change. Many of the mothers in our groups have had a long experience of being in therapy. They were all well-known to the welfare services and had extensive experience in telling their narratives in a therapeutic setting. Drama therapy, however, encouraged a different type of involvement with their personal narrative about themselves, their children, and their relationships. The opportunity to embody, to role-play, and to project provided a unique opportunity and served as a crucial step toward change, especially in a population that is “over-served” in therapeutic encounters.

## Limitations

The absence of a control group makes it difficult to draw inferences about the particular components that contributed to the therapeutic change, whether it was passage of time, the development of the child or other variables that are not directly connected to the therapeutic model we examined. In addition, further exploration is needed to catalyze what is the specific component of the therapeutic model we offered that enhanced the possibility for a therapeutic change. For example, whether it was the drama that made the change possible, the qualities of the group format (i.e., universality, validation, being witnessed, and social support), the fact that it was a short-term-focused intervention, or some other component of the therapeutic encounter.

Another question that remains unanswered is the change in children’s behavior problems that manifested mostly as externalized problems and less in the internalized ones. One explanation may suggest that better maternal insightfulness leads to the feeling of children being more understood by their mothers and that in turn, reduces the need to use externalized behavior to communicate their feeling. Interestingly, overall, in studies of parent-child relations, externalizing behavior is the most frequently investigated type of child behavior, with fewer studies and smaller effect on internalizing problems (Klein Velderman et al., 2006). This might be due to the fact that changes in externalizing problems are more easily observed.

Future studies, using a larger sample, need to explore further this behavioral change and to collect behavioral reports from sources other than the mothers (for example, teachers) to obtain a richer and more valid account. Another point to be taken is the age range of children in our study that was quite wide (7–12), future studies may focus on a more narrow age range.

To summarize, the results of the study reinforced the idea that drama therapy for parents can be engaging, accessible, and effective. The study attests to the importance of using conceptualized thinking to create a manualized treatment and contribute to evidence-based practice in drama therapy. We hope this study broadens the dialogue between theory, research, and clinical work.

## Data Availability Statement

The raw data supporting the conclusions of this article will be made available by the authors, without undue reservation.

## Ethics Statement

The studies involving human participants were reviewed and approved by University of Haifa IRB committee. The patients/participants provided their written informed consent to participate in this study.

## Author Contributions

RF-S was the main researcher who initiated and designed the study, supervise the data collection, designed the intervention, and supervised the clinical team. NK-K contributed to the design of the study, analyzed all the Insightfulness assessment interviews, supervised some of the research assistance in the study, and contributed to the conceptualization of the study. Both were involved in the data analysis and the final writing. Both the authors contributed to the article and approved the submitted version.

### Conflict of Interest

The authors declare that the research was conducted in the absence of any commercial or financial relationships that could be construed as a potential conflict of interest.

## Footnotes

^1^Because the focus of the project described in this paper were mothers, we use the term *maternal insightfulness* to describe the concept in relation to the data we collected.

^2^When calculating ICC, 0.5 are indicative of poor reliability, values between 0.5 and 0.75 indicate moderate reliability, values between 0.75 and 0.9 indicate **good** reliability, and values greater than 0.90 indicate excellent.

^3^Kappa result to be interpreted as follows: values ≤0 as indicating no agreement and 0.01–0.20 as none to slight, 0.21–0.40 as fair, 0.41–0.60 as moderate, 0.61–0.80 as substantial, and 0.81–1.00 as almost perfect agreement.
