# Supplementary material for: Using Drama Therapy to Enhance Maternal Insightfulness and Reduce Children’s Behavior Problems
Source: Front Psychol. 2021 Jan 20;11:586630. doi: 10.3389/fpsyg.2020.586630 (PMC7854457; doi:10.3389/fpsyg.2020.586630)
Supplement: Supplementary file 1 [file Table_1.docx]

| Maternal insightfulness scales | Time of assessment | *M* | *SD* | *t* | *P* |
| --- | --- | --- | --- | --- | --- |
| Focus | T1  T3 | 4.88  6.40 | 1.89  2.11 | -3.560 | .001 |
| Insight | T1  T3 | 4.20  5.32 | 1.41  1.53 | -4.140 | .000 |
| Acceptance | T1  T3 | 4.48  5.84 | 1.86  2.03 | -3.384 | .002 |
| Hostility | T1  T3 | 2.52  1.87 | 1.90  1.28 | 1.970 | .058 |
| Concern | T1  T3 | 5.15  3.26 | 2.20  2.56 | 4.041 | .000 |
| Separateness | T1  T3 | 6.51  7.47 | 1.73  1.70 | -2.330 | .026 |
| Openness | T1  T3 | 5.02  6.02 | 1.73  1.79 | -2.827 | .008 |
| Richness | T1  T3 | 4.38  5.59 | 1.44  1.62 | -3.576 | .001 |
| Coherence | T1  T3 | 3.88  5.44 | 1.38  1.77 | -5.172 | .000 |
| Complexity | T1  T3 | 4.73  6.19 | 1.73  1.65 | -4.175 | .000 |

Difference in maternal insightfulness scales between T1 and T3
